# Supplementary material for: Supraspecific units in correlative niche modeling improves the prediction of geographic potential of biological invasions
Source: PeerJ. 2020 Dec 22;8:e10454. doi: 10.7717/peerj.10454 (PMC7761189; doi:10.7717/peerj.10454)
Supplement: Supplemental Information 1 [file peerj-08-10454-s001.pdf]

Supplemental information

Table S1: Input data for the ecological niche models. NSp = number of species included in the modeling unit. MVE = minimum-volume ellipsoid.

Focal species	Unit name	NSp	Number of presence records used in MVE modeling		Number of presence records used in Maxent modeling
			95%	97.50%	
Aedes aegypti	U0	1	484	490	196
	U1	2	405	444	579
	U2	5	701	706	746
	U3	8	1855	1903	1470
	U4	9	1987	2008	1587
	UT	1	29940	30415	2866
Pterois volitans	U0	1	389	390	80
	U1	2	493	503	80
	U2	4	788	793	80
	U3	5	876	881	100
	UT	1	643	651	101
Orechromis mossambicus	U0	1	362	362	98
	U1	2	596	599	185
	U2	3	682	686	194
	U3	5	612	619	198
	U4	6	525	527	201
	U5	8	750	754	262
	UT	1	718	724	783

Table S2: Environmental surfaces used in the estimation of the MVEs of the terrestrial species.

Bioclimatic variables	Bioclimatic variables full names
BIO1	Annual mean temperature (°C)
BIO2	Mean diurnal temperature range (mean (period max - min)) (°C)
BIO3	Isothermality (BIO2/BIO7)
BIO4	Temperature seasonality (C of V)
BIO5	Max temperature of warmest week (°C)
BIO6	Min temperature of coldest week (°C)
BIO7	Temperature annual range (BIO5-BIO6) (°C)
BIO10	Mean temperature of warmest quarter (°C)
BIO11	Mean temperature of coldest quarter (°C)
BIO12	Annual precipitation (mm)
BIO13	Precipitation of wettest week (mm)
BIO14	Precipitation of driest week (mm)
BIO16	Precipitation of wettest quarter (mm)
BIO17	Precipitation of driest quarter (mm)
BIO20	Annual mean radiation (W m-2)
BIO21	Highest weekly radiation (W m-2)
BIO22	Lowest weekly radiation (W m-2)
BIO23	Radiation seasonality (C of V)
BIO28	Annual mean moisture index
BIO29	Highest weekly moisture index
BIO30	Lowest weekly moisture index
BIO32	Mean moisture index of wettest quarter
BIO33	Mean moisture index of driest quarter

Table S3: Environmental surfaces used in the estimation of the MVEs of the marine species. Lt: average of minimum and maximum records per year.

**Bioclimatic variables**
Present Surface Temperature Min (°C)
Present Surface Temperature Max (°C)
Present Surface Temperature Mean (°C)
Present Surface Temperature Lt Min (°C)
Present Surface Temperature Lt Max (°C)
Present Surface Temperature Range (°C)
Present Surface Salinity Min (PSS)
Present Surface Salinity Max (PSS)
Present Surface Salinity Mean (PSS)
Present Surface Salinity Lt Min (PSS)
Present Surface Salinity Lt Max (PSS)
Present Surface Salinity Range (PSS)

Maxent methods

Maxent models were calibrated across the **M** area designed for each modeling unit. We partitioned presence records into two sets, training and evaluation, using the block function of ENMeval (Muscarella et al., 2014). Settings for the model construction were: the crossvalidation/replicated functionality, three features (l=linear, q=quadratic and p=product), regularization multipliers from 1 up to 4, and a “logistic” output. We chose the previous features trying to emulate mathematical functions fitted in MVEs, in order to minimize differences between algorithms given by factors other than the presence data. We also set aside 50% of presences as a test percentage and conducted five replicates analysis to take into account the variances due to specific calibration data sets on model outputs. Finally, the models were projected worldwide applying truncation as transfer procedure.

Maxent results

In all three species, the AUC ratio of the partial ROC test increased when occurrences of closely related species were included (Fig. S3). This increase was similar to that observed in the MVEs, where it was clearer in *Ae. aegypti* and *P. volitans* whereas in *O. mossambicus* the greatest increase in performance was reached up to U5.

For *Ae. aegypti*, thresholded models of U0 failed to predict the invasion in different regions that are predicted by U1, such as in the Florida Peninsula, central Mexico, the Atlantic Forest and eastern Australia (Fig. S4B and Fig. S5A and C).

In *P. volitans*, unlike U0, the U1 model predicts invasion in the Red Sea, the Adriatic Seas and in the Persian Gulf (Fig. S4D and Fig. S5D and E).

The U0 model of *O. mossambicus* predicts regions with invasion records in Florida, Texas, central Mexico and eastern Australia that correspond to areas not predicted by U5. In contrast, the U5 model predicts the invasion locations in part of Central America, the Mexican Pacific, the Colombian Andes, Madagascar, and Southeast Asia that are not predicted in thresholded models of U0 (Fig. S4F and Fig. S5G and I).

Supplemental figure legends

Figure S1: Scree plot showing the decreasing rate at which variance is explained by additional principal components of the terrestrial environment.

Figure S2: Scree plot showing the decreasing rate at which variance is explained by additional principal components of the marine environment.

Figure S3: Average AUC ratios of the ecological niche models obtained with Maxent for each modeling unit. Bars indicate the standard deviation.

Figure S4: Presence records of the native range (black X's), invasion range (green X's), and those included in the supraspecific unit with the highest AUC ratio (red X's) of *Aedes aegypti* (A), *Pterois volitans* (C) and *Oreochromis mossambicus* (E). Potential distribution models obtained with Maxent using the black X's as input presence data (light blue) (i.e., U0), and potential distribution models obtained with Maxent using the black X's + red X's as input presence data (dark blue) (i.e., U1) for *Ae. aegypti* (B), *P. volitans* (D) and *O. mossambicus* (F).

Figure S5: Regional views of the potential distribution (obtained with Maxent) across the invasion area of *Aedes aegypti* (A, B and C). *Pterois volitans* (D, E and F) and *Oreochromis mossambicus* (G, H and I). Light blue model = distribution estimated from U0; Dark blue model = distribution estimated from the supraspecific unit with the highest AUC ratio; Green X's = presence records of the invaded areas.
