# Supplementary figures and images for: Supraspecific units in correlative niche modeling improves the prediction of geographic potential of biological invasions
Source: PeerJ. 2020 Dec 22;8:e10454. doi: 10.7717/peerj.10454 (PMC7761189; doi:10.7717/peerj.10454)

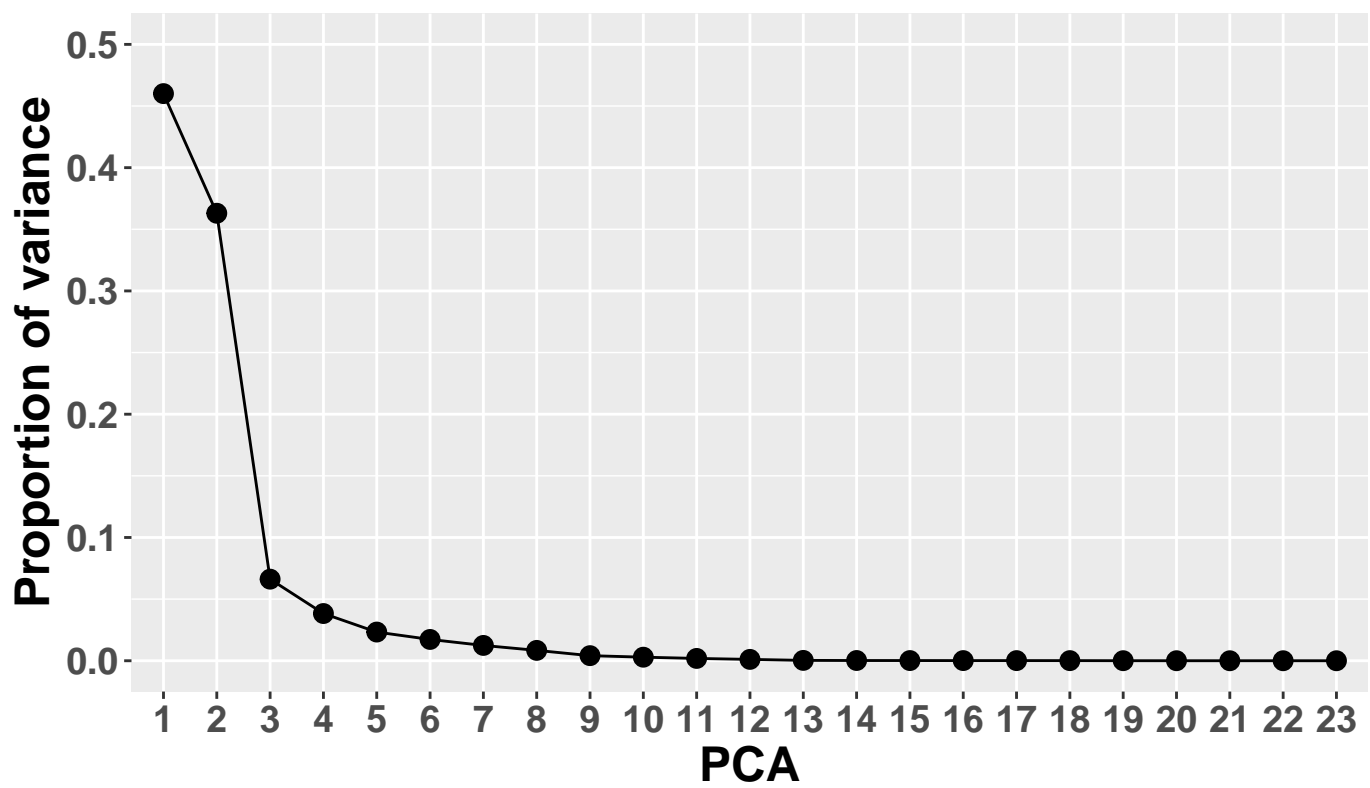

Supplement: Supplemental Information 2 [file peerj-08-10454-s002.pdf]

**Proportion of variance**

0.6  
0.4  
0.2  
0.0

1

2

3

4

5

6

7

8

9

10

11

12

**PCA**

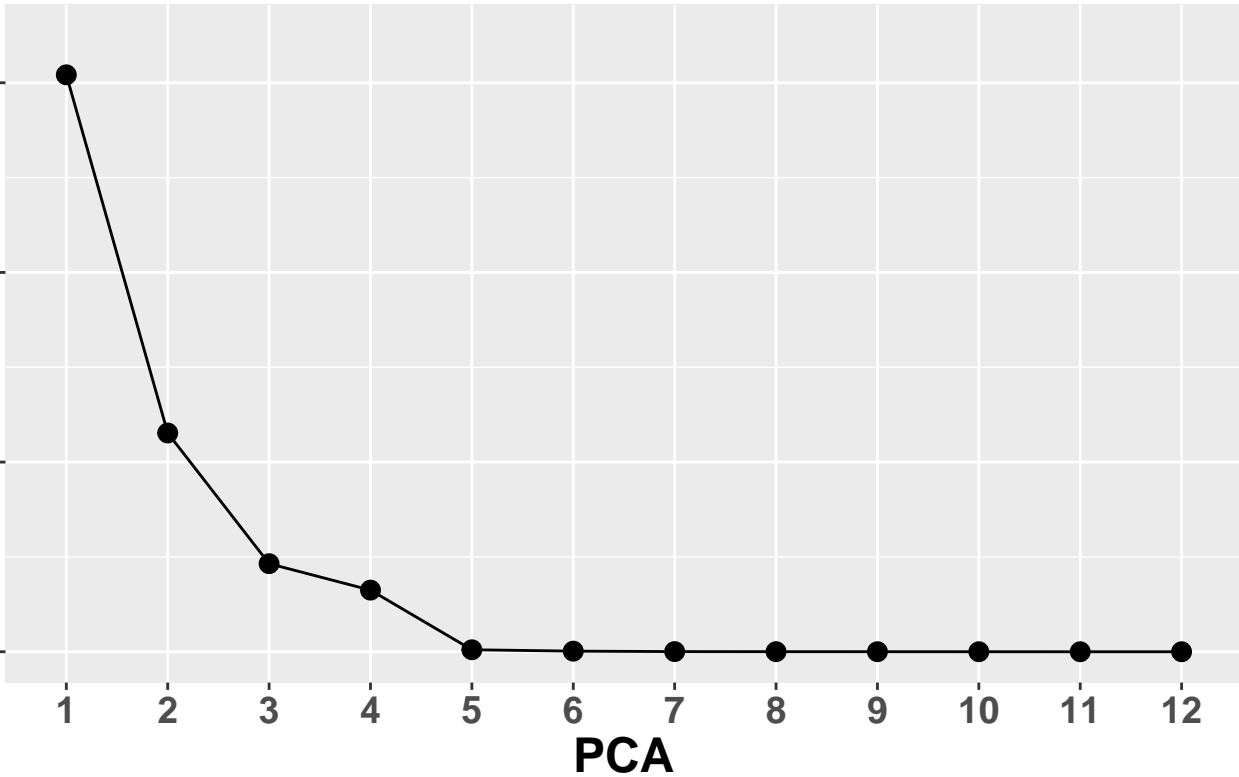

Supplement: Supplemental Information 3 [file peerj-08-10454-s003.pdf]

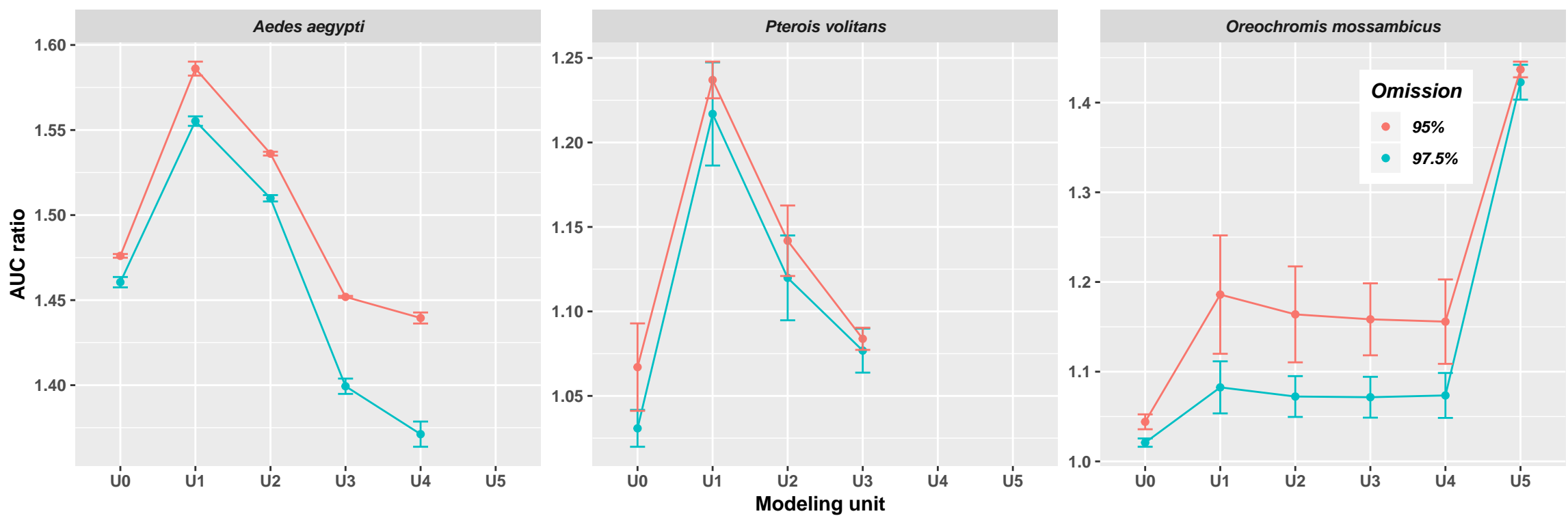

Supplement: Supplemental Information 4 — Bars indicate the standard deviation. [file peerj-08-10454-s004.pdf]

**A**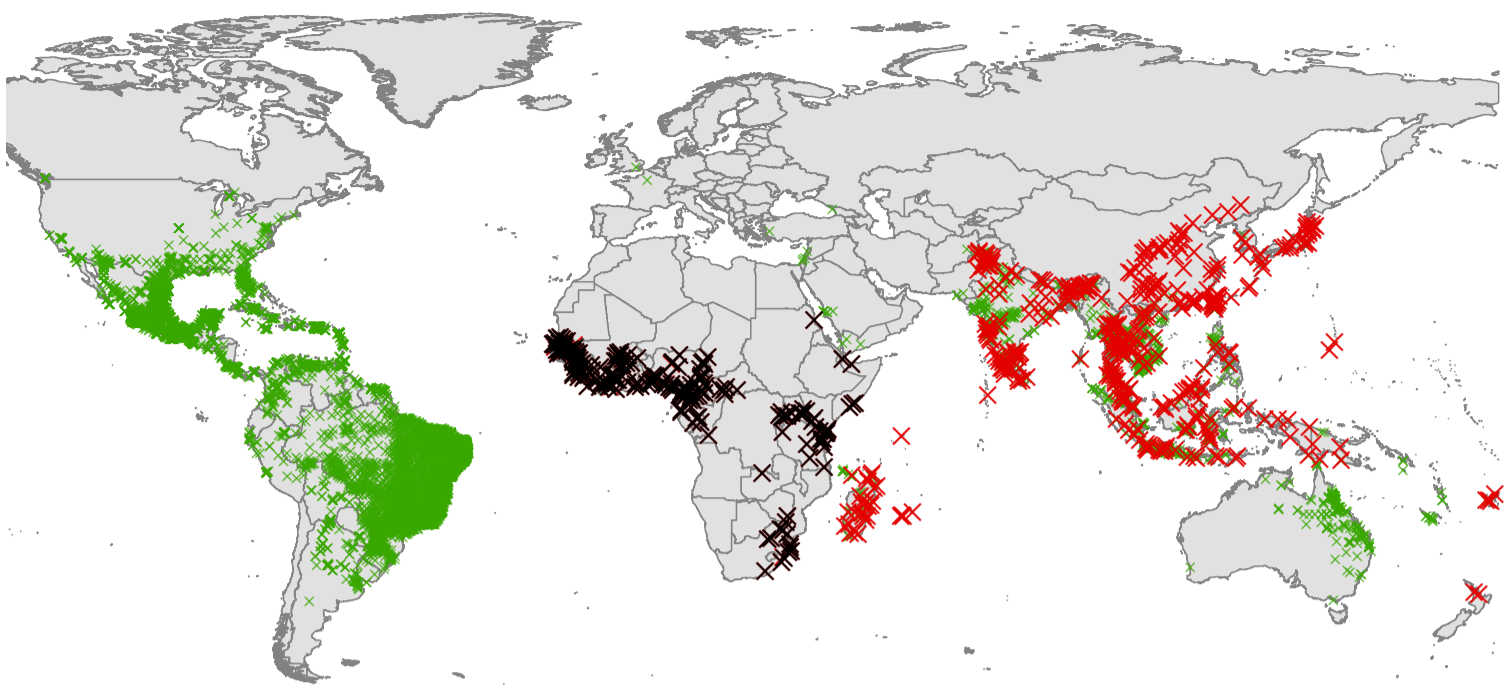**B**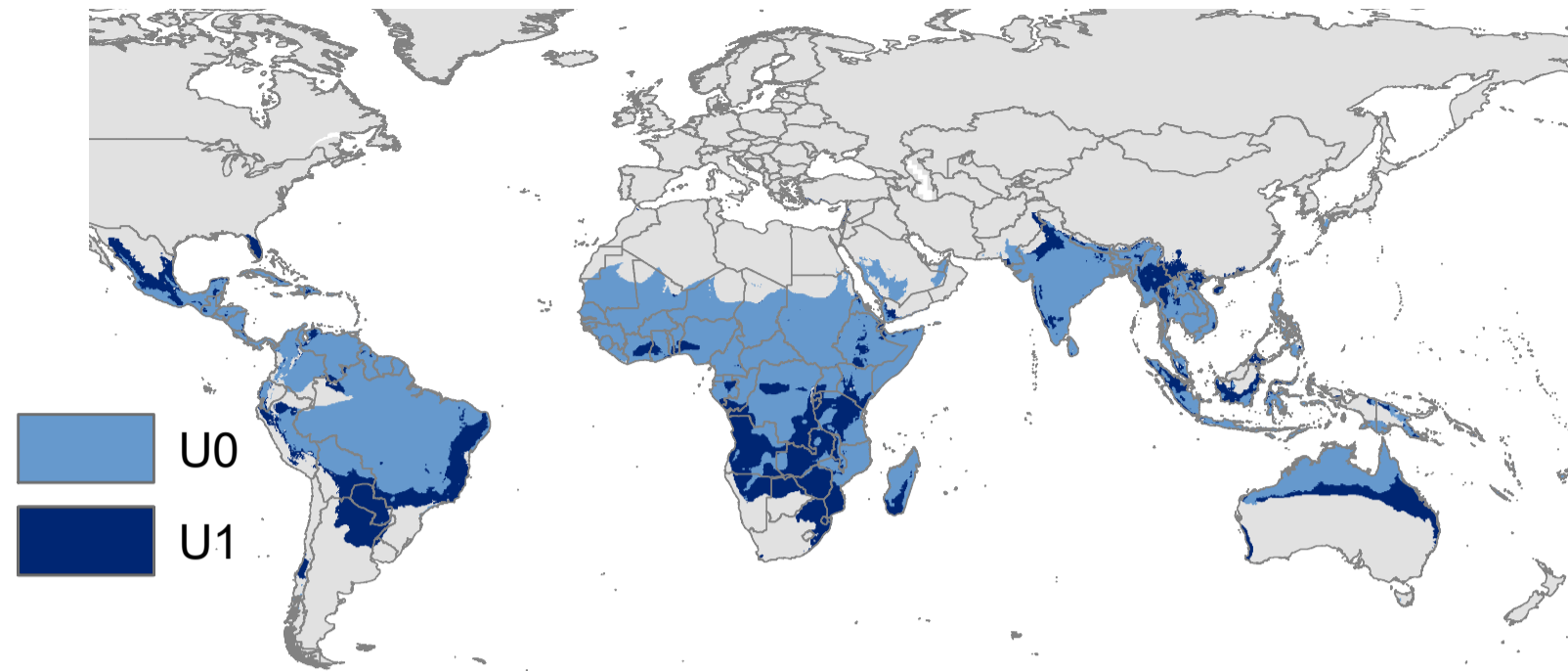**C**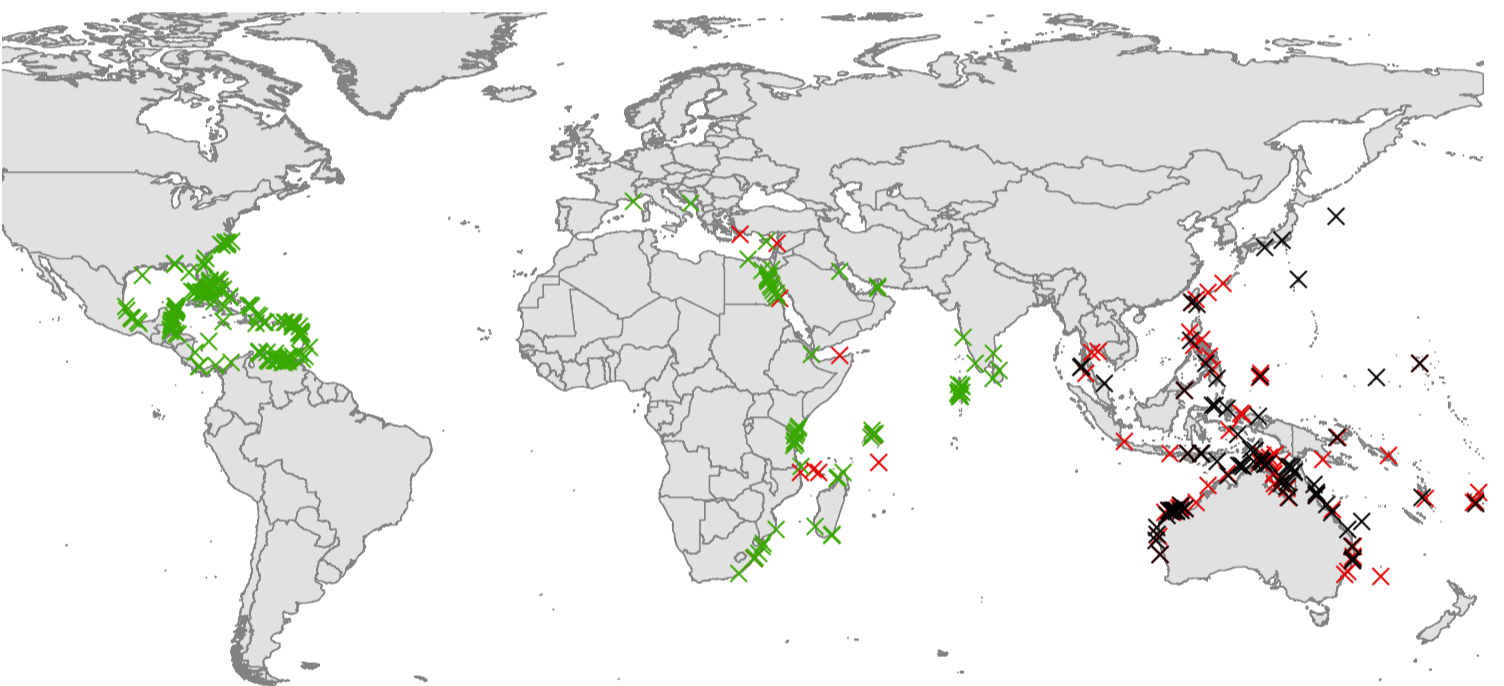**D**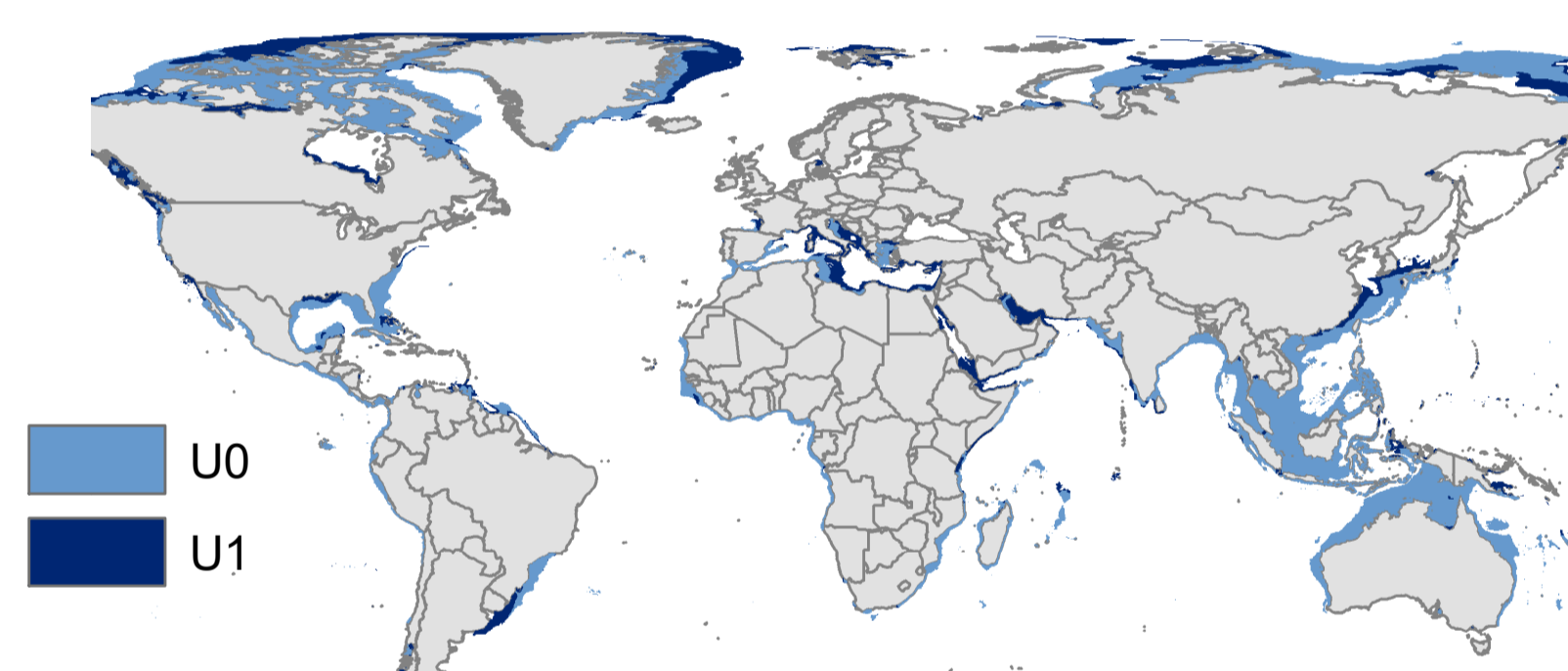**E**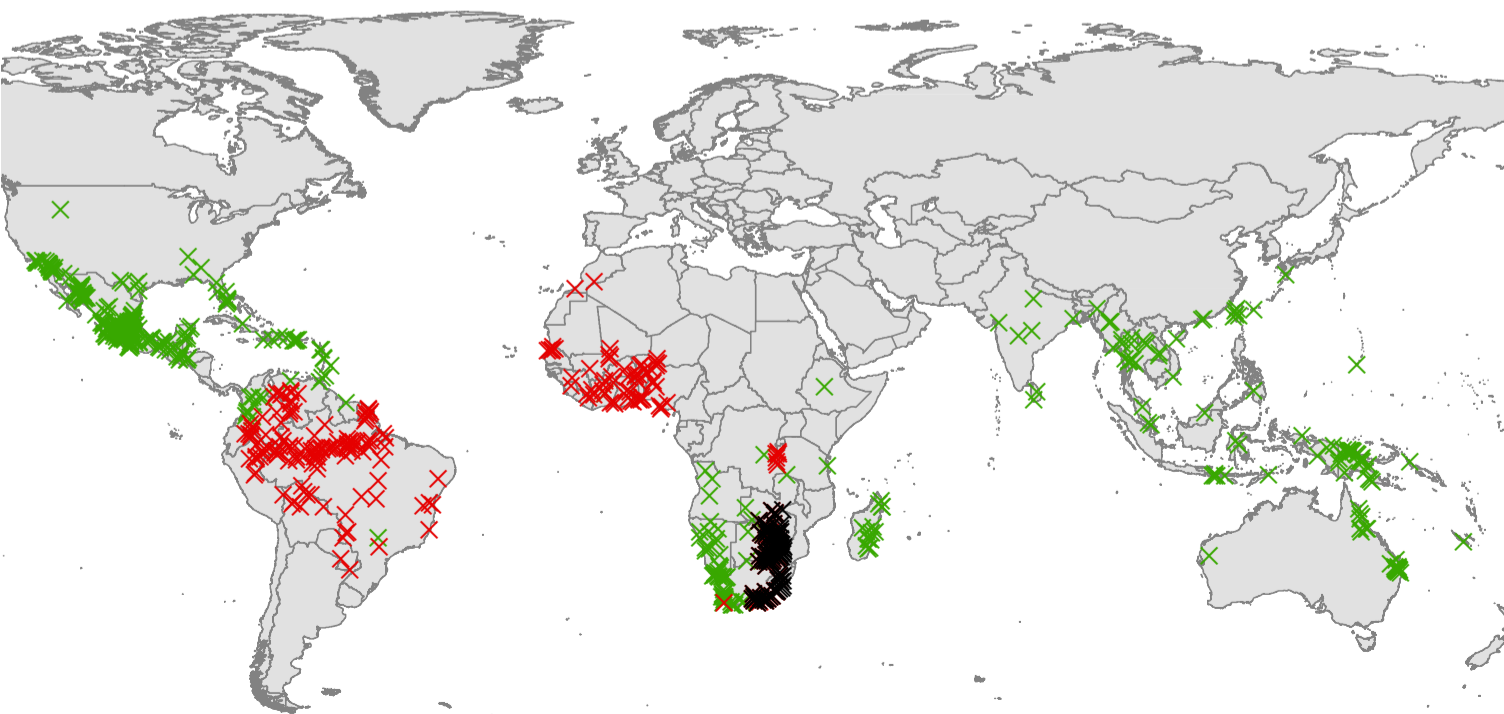**F**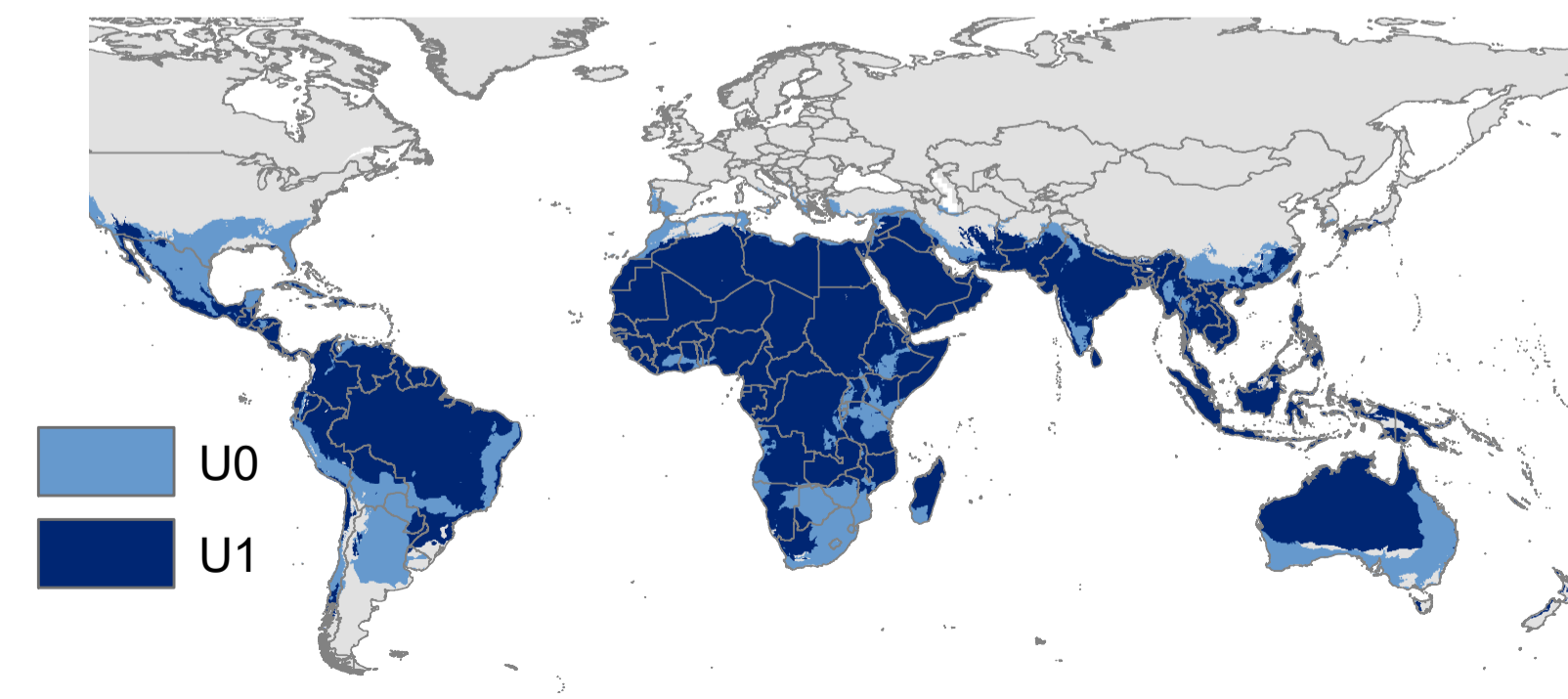

Supplement: Supplemental Information 5 — Presence records of the native range (black X’s), invasion range (green X’s), and those included in the supraspecific unit with the highest AUC ratio (red X’s) of Aedes aegypti (A), Pterois volitans (C) and Oreochromis mossambicus (E). Potential distribution models obtained with Maxent using the black X’s as input presence data (light blue) (i.e., U0), and potential distribution models obtained with Maxent using the black X’s + red X’s as input presence data (dark blue) (i.e., U1) for Ae. aegypti (B), P. volitans (D) and O. mossambicus (F). [file peerj-08-10454-s005.pdf]

**A**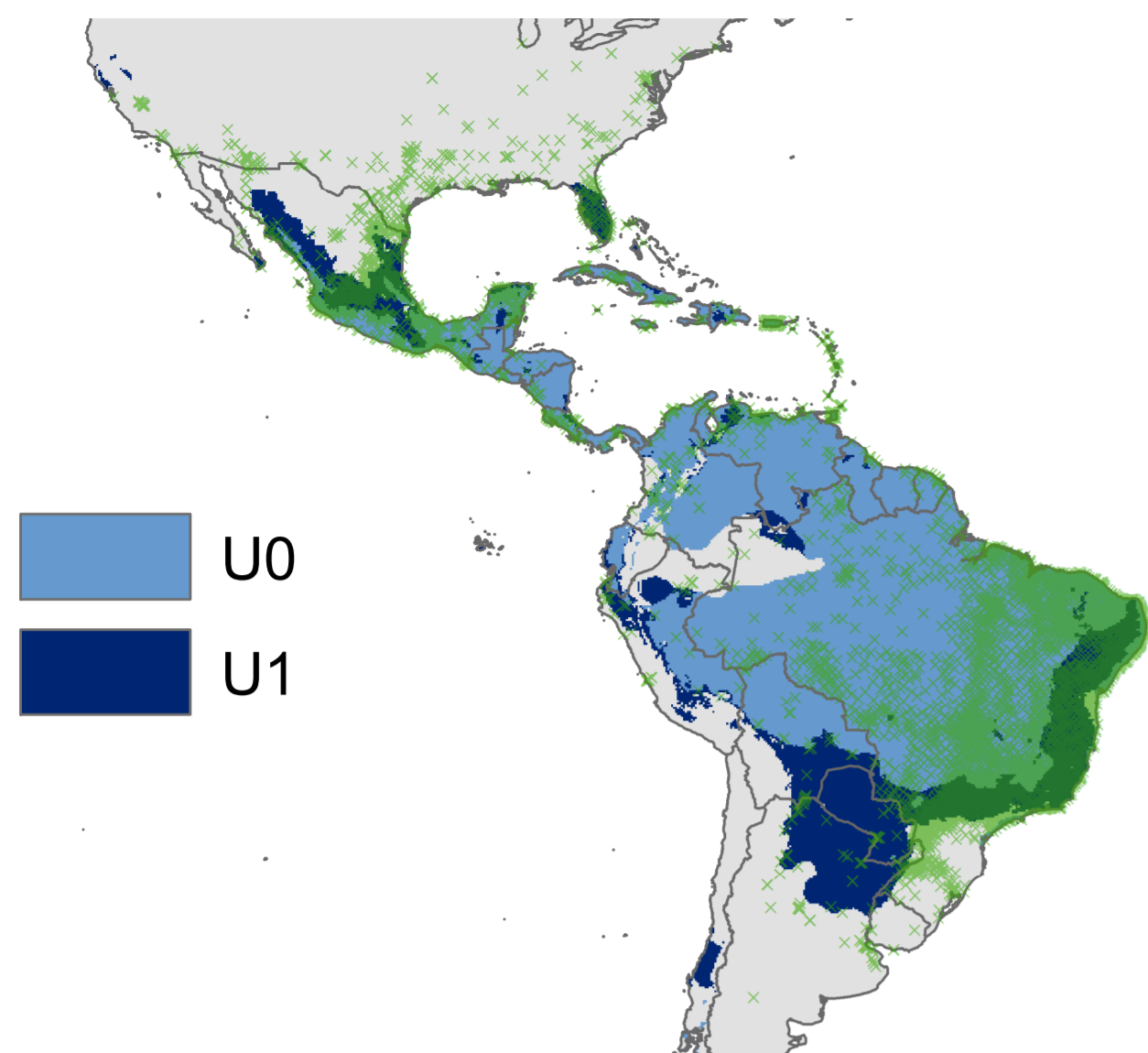**B**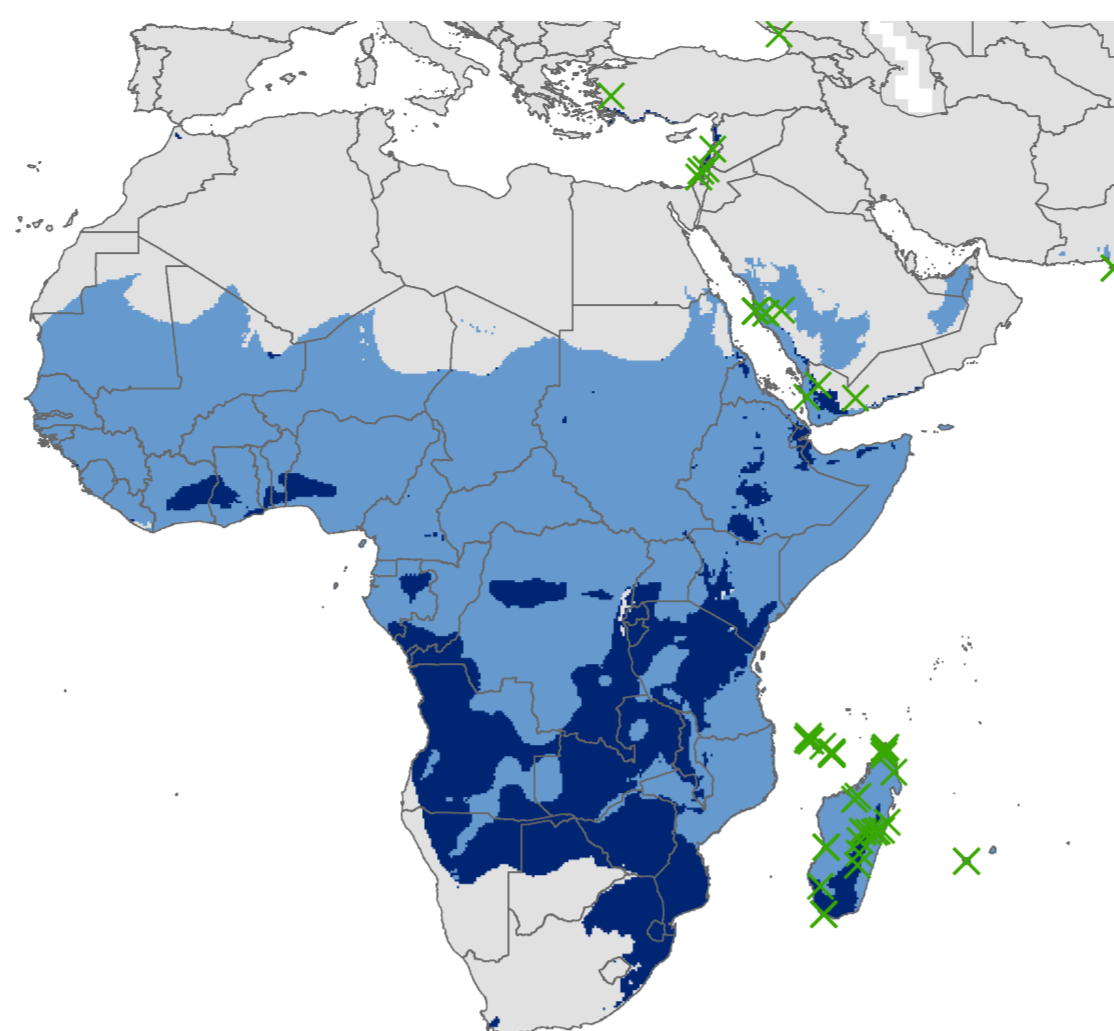**C**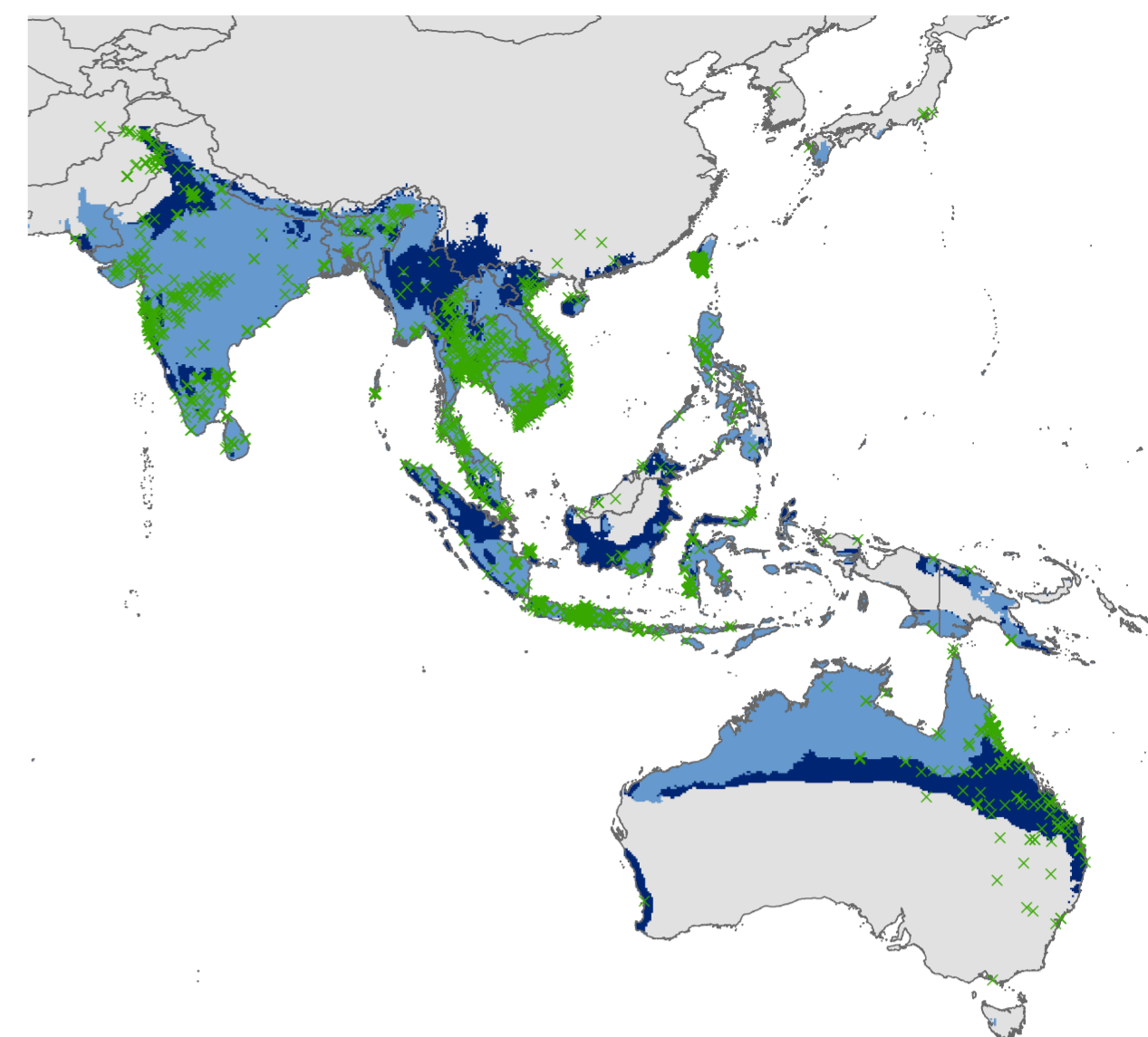**D**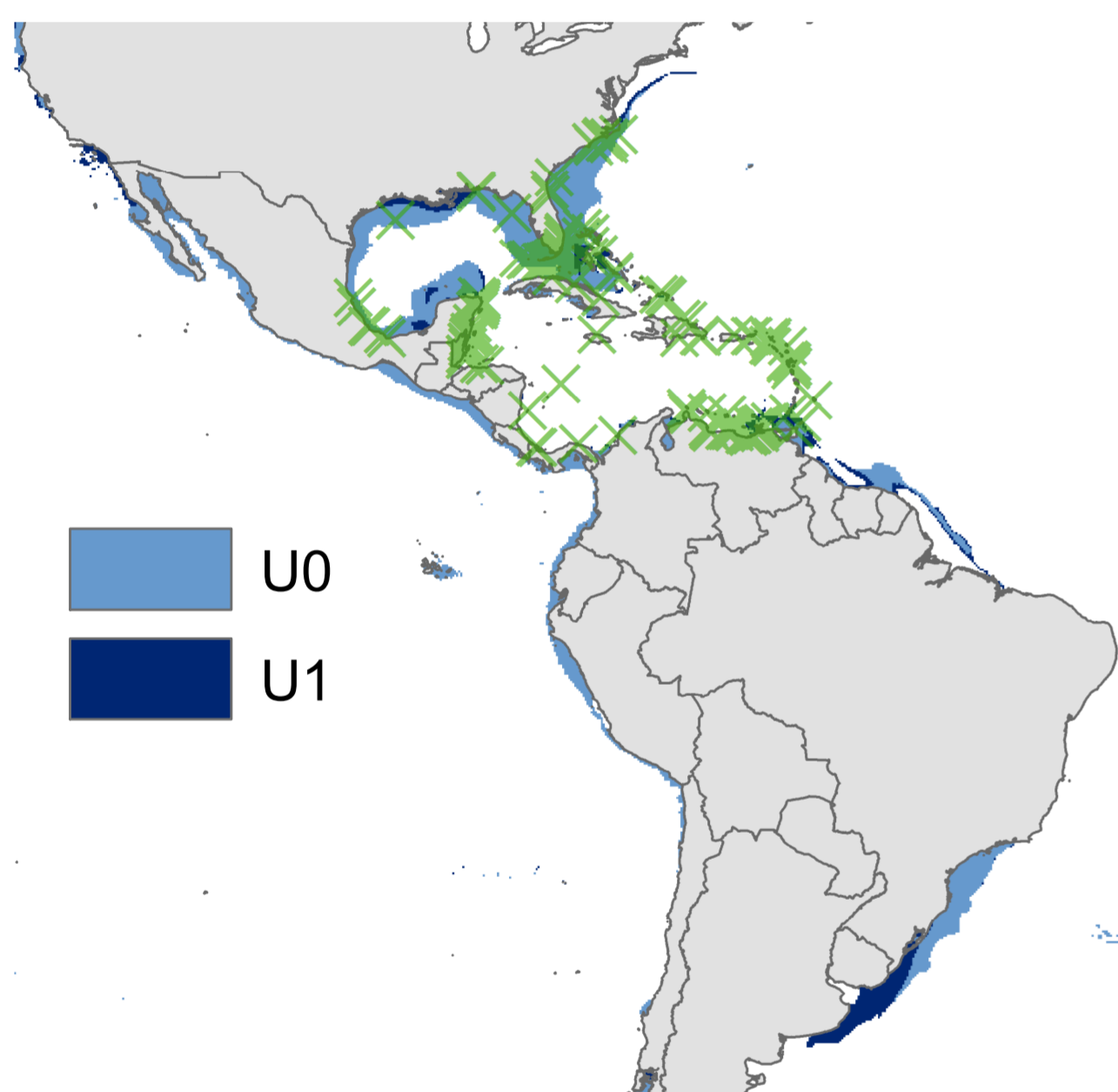**E**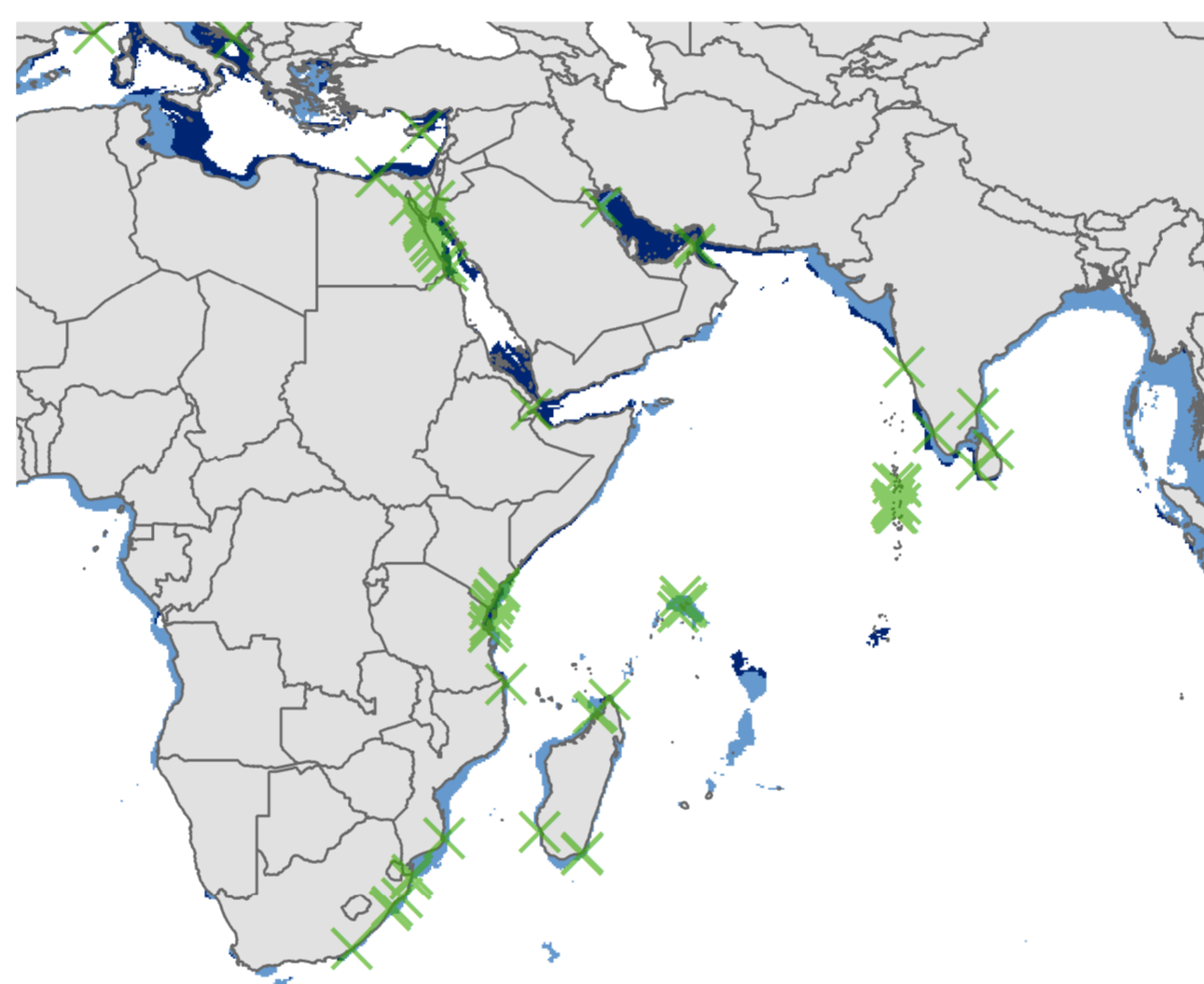**F**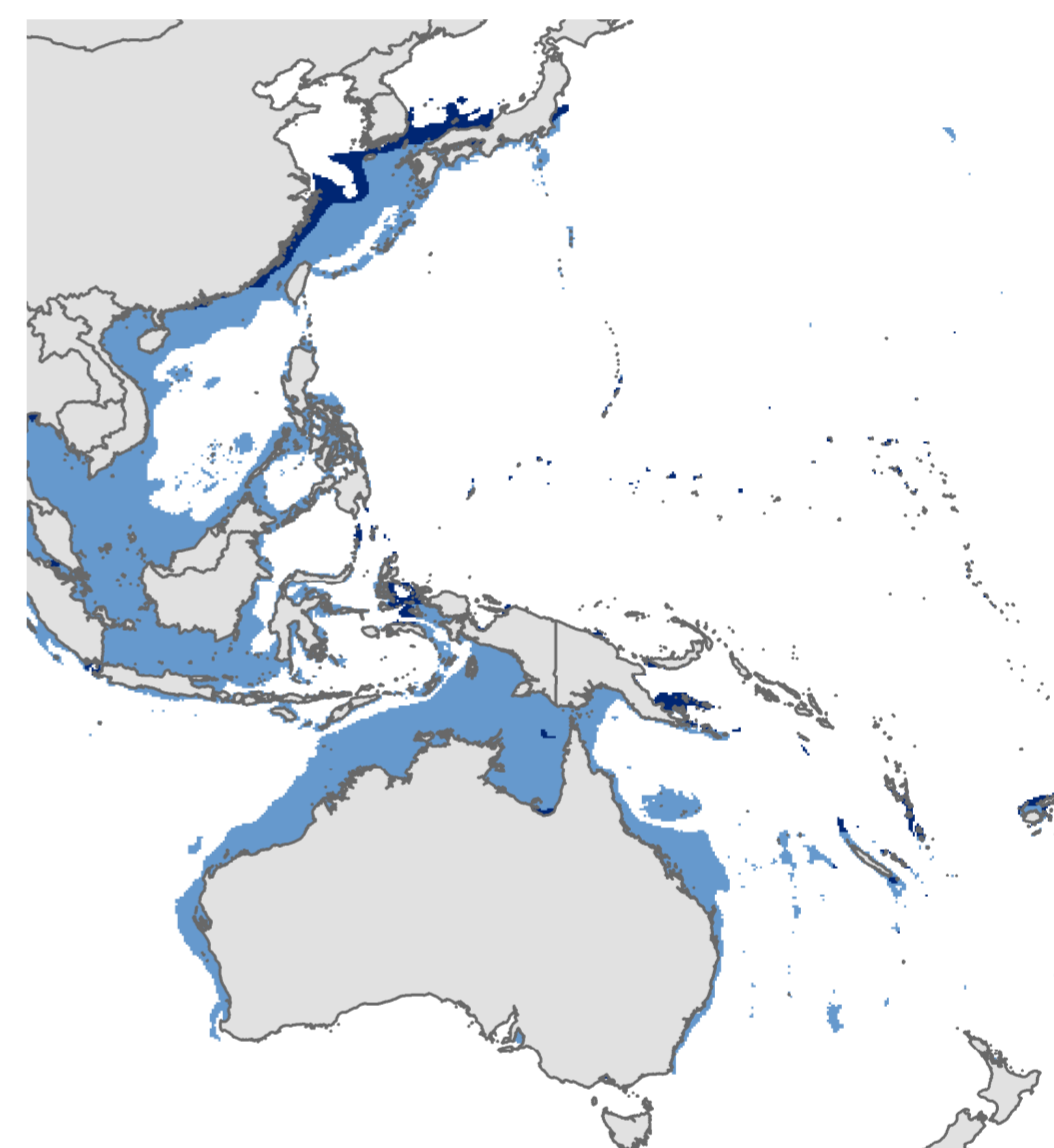**G**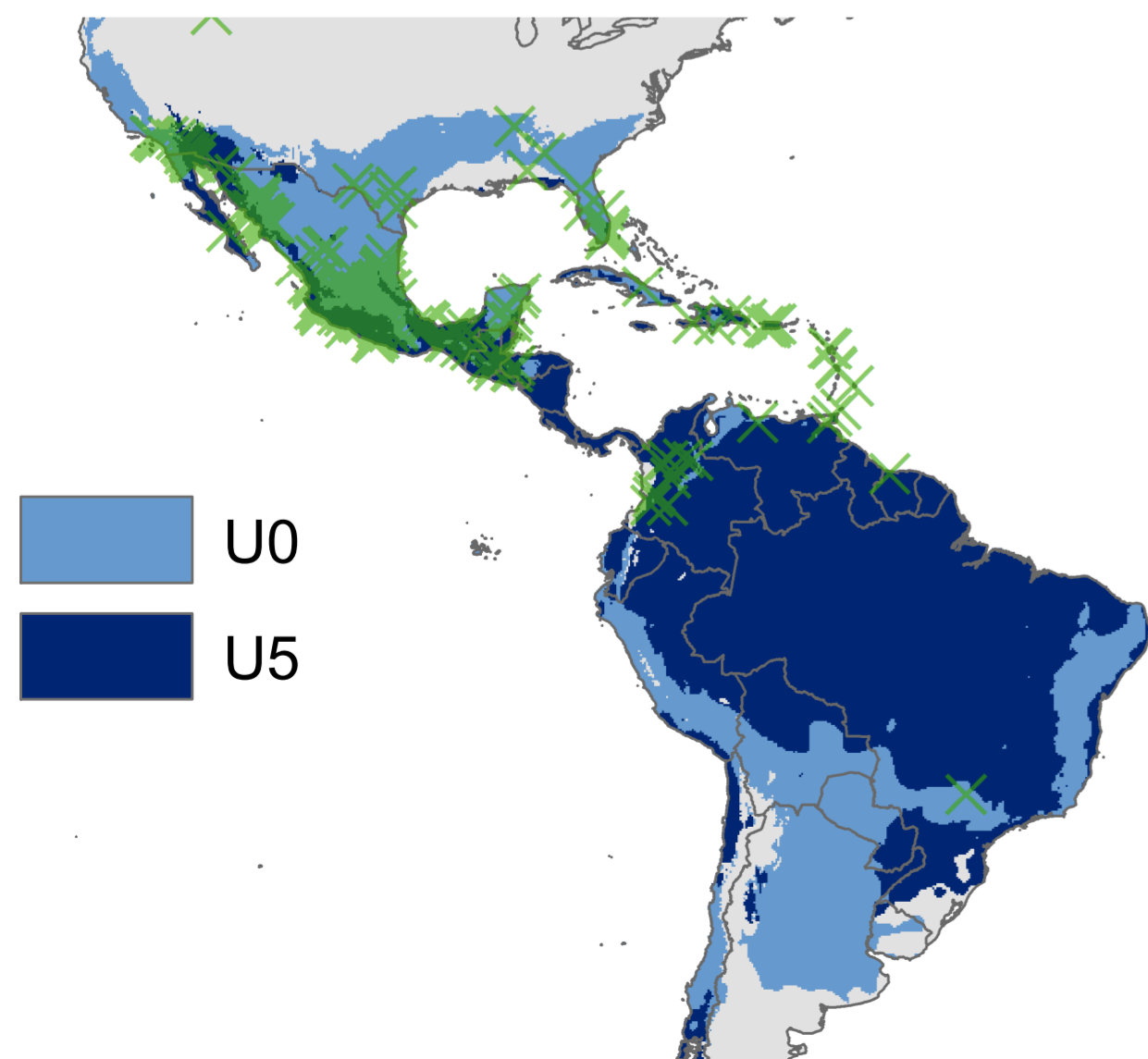**H**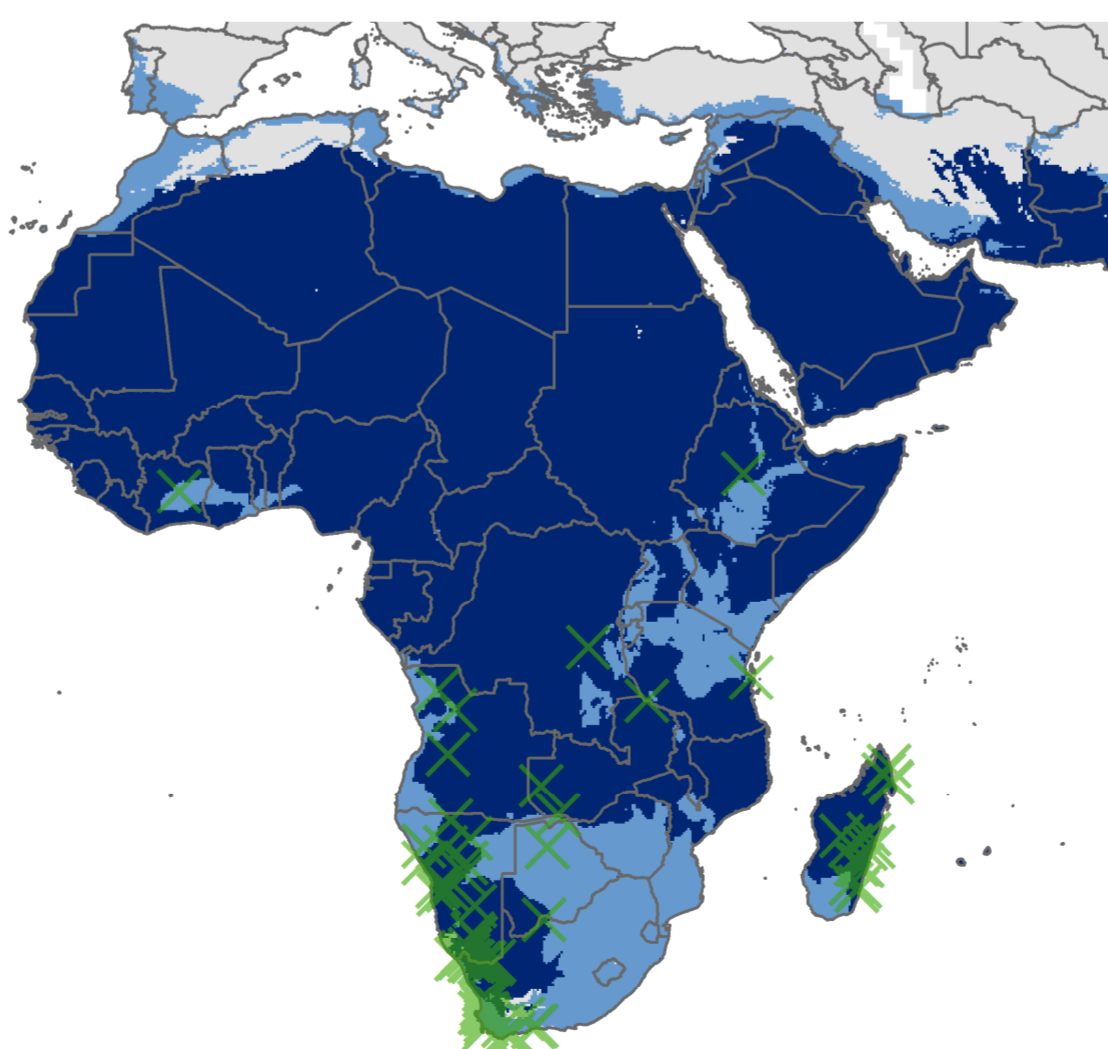**I**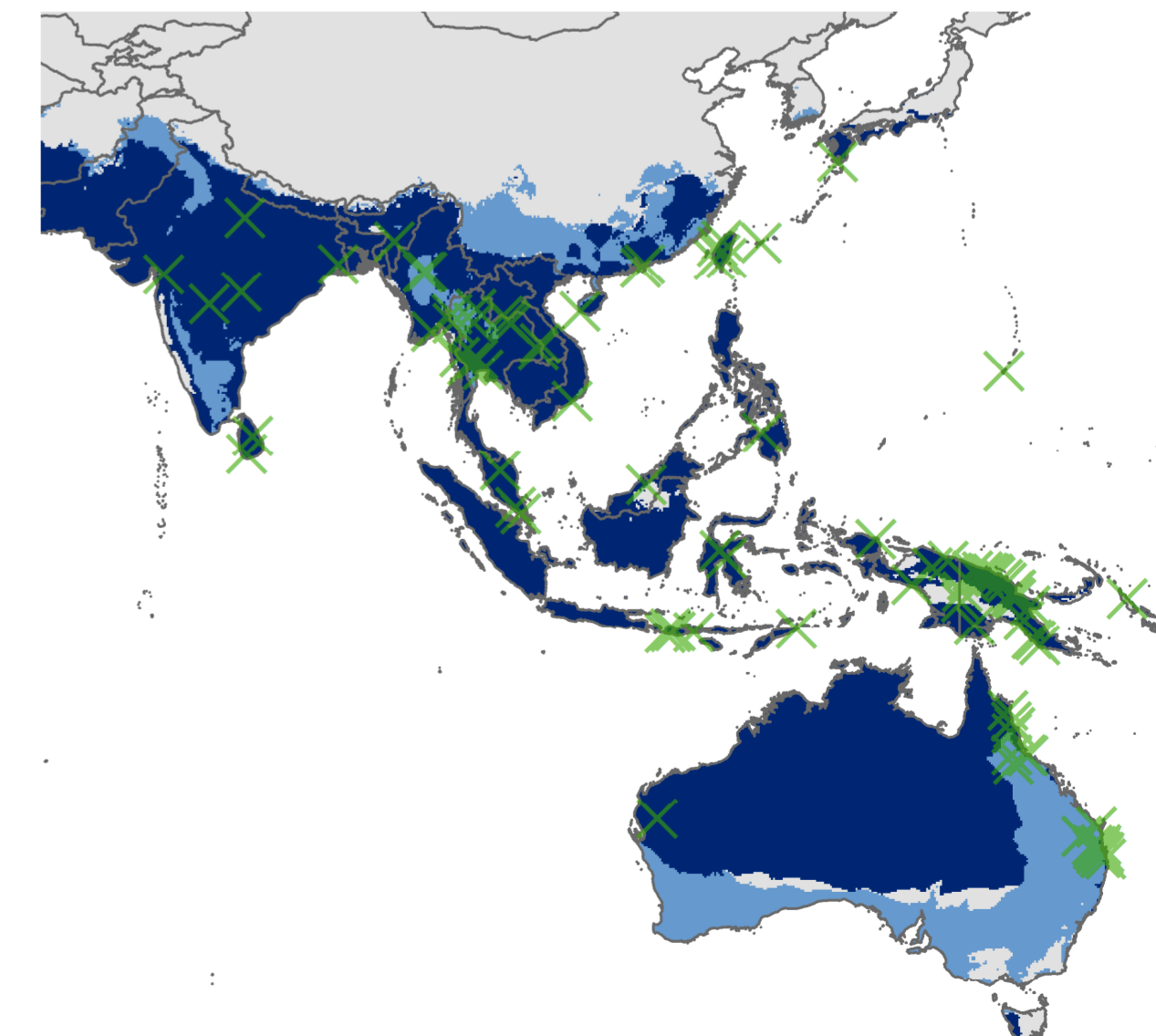

Supplement: Supplemental Information 6 — Light blue model = distribution estimated from U0; Dark blue model = distribution estimated from the supraspecific unit with the highest AUC ratio; Green X’s = presence records of the invaded areas. [file peerj-08-10454-s006.pdf]
